# RNA helicase p68 inhibits the transcription and post-transcription of *Pkd1* in ADPKD

**DOI:** 10.7150/thno.47315

**Published:** 2020-07-09

**Authors:** Lu Zhang, Linda Xiaoyan Li, Julie Xia Zhou, Peter C. Harris, James P. Calvet, Xiaogang Li

**Affiliations:** 1 Department of Internal Medicine and Biochemistry and Molecular Biology, Mayo Clinic, Rochester, MN 55905.; 2 Department of Nephrology, Renmin Hospital of Wuhan University, Wuhan 430060, China.; 3 Department of Biochemistry and Molecular Biology, University of Kansas Medical Center, Kansas City, KS 66160.

**Keywords:** p68, transcription regulation, miRNA, *PKD1*, ADPKD

## Abstract

**Background:** Autosomal dominant polycystic kidney disease (ADPKD) is caused by mutations of the *PKD1* and *PKD2* genes. Dysregulation of the expression of PKD genes, the abnormal activation of PKD associated signaling pathways, and the expression and maturation of miRNAs regulates cyst progression. However, the upstream factors regulating these abnormal processes in ADPKD remain elusive.

**Methods:** To investigate the roles of an RNA helicase, p68, in ADPKD, we performed Western blot and qRT-PCR analysis, immunostaining and ChIP assay in cystic renal epithelium cells and tissues.

**Results:** We found that p68 was upregulated in cystic renal epithelial cells and tissues. p68 represses *Pkd1* gene expression via transcriptional and posttranscriptional mechanisms in renal epithelial cells, in that 1) p68 binds to the promoter of the *Pkd1* gene together with p53 to repress transcription; and 2) p68 promotes the expression and maturation of miR-17, miR-200c and miR-182 and via these miRNAs, post-transcriptionally regulates the expression of *Pkd1* mRNA. Drosha is involved in this process by forming a complex with p68. p68 also regulates the phosphorylation and activation of PKD proliferation associated signaling and the expression of fibrotic markers in *Pkd1* mutant renal epithelial cells. Silence of p68 delays cyst formation in collecting duct cell mediated 3D cultures. In addition, the expression of p68 is induced by H_2_O_2_-dependent oxidative stress and DNA damage which causes downregulation of *Pkd1* transcription in cystic renal epithelial cells and tissues.

**Conclusions:** p68 plays a critical role in negatively regulating the expression of the *PKD1* gene along with positively regulating the expression and maturation of miRNAs and activation of PKD associated signaling pathways to cause renal cyst progression and fibrosis in ADPKD.

## Introduction

Autosomal dominant polycystic kidney disease (ADPKD) is among the most common monogenic human disorders. It is characterized by the progressive development of fluid-filled cysts in the kidney, liver and pancreas and is associated with hypertension, kidney failure and brain aneurysms 1, 2. ADPKD occurs equally in men and women, across all ethnic groups, and affects over 12 million people worldwide. Mutations of one or more PKD genes, including *PKD1* (in 78% of disease pedigrees), *PKD2* (in 13% of disease pedigrees) and GANAB (in ~0.3% of disease pedigrees) result in cyst formation 3. The severity of ADPKD is associated with large interfamilial and intrafamilial variability, which may be explained in part by genetic heterogeneity, epigenetic modification and transcriptional regulation of PKD gene expression. The *PKD1* gene encodes a large protein, polycystin-1 (PC1), which forms multiprotein complexes at the cell membrane and primary cilia to regulate cell-cell and cell-matrix interactions, signal transduction, and mechanosensation. It has been found that expression of the *PKD1* gene under a critical threshold can result in cystogenesis 4. However, the factors and mechanisms that regulate the transcription of the PKD genes remain largely unknown.

The p68 RNA helicase (also called DEAD-box protein 5; DDX5) is a prototypic member of the DEAD box family of RNA helicases that exhibits ATPase and RNA unwinding activities 5. The DEAD box family is named after the amino acid sequence of its conserved Motif II (also known as the Walker B motif) containing the amino acids asp-glu-ala-asp (D-E-A-D). As one of the first DEAD-box family proteins to exhibit RNA helicase activity, p68/DDX5 plays an important role in the regulation of gene transcription, cell proliferation, early organ development and maturation, and DNA damage repair pathways 6. In addition, p68 plays an apparently RNA helicase-independent role as a transcriptional co-activator of several cancer-associated transcription factors, including β-catenin, p53, estrogen receptor α, and androgen receptor 7. As a transcriptional co-activator, p68 can be recruited to the promoters of its target genes together with the activated transcription factors. For example, p68 is selectively required for the induction of p53-dependent p21 expression by promoting the recruitment of p53 and RNA polymerase II to the CDKN1 (p21) promoter 8, resulting in cell-cycle arrest after DNA damage. p68 also plays a crucial role as a selective factor that favors p53-mediated growth arrest and is required for the induction of apoptosis, both in cultured cells and *in vivo* 9. Recent studies have demonstrated that p68 is abnormally expressed in several types of cancers. Overexpression of p68 leads to the development of colorectal tumors, breast cancer, leukemia, and prostate cancer, suggesting that p68 plays critical roles in cancer development and progression 10. However, the expression of p68 in ADPKD and the roles of p68 in PKD gene transcription and cystic renal epithelial cell proliferation have not been explored.

The identification of p68 as a component of the Drosha complex also highlights its potential involvement in microRNA (miRNA) biogenesis 11. It has been shown that p68-null mouse embryonic fibroblasts (MEFs) are defective in the processing of a subset of primary (pri)-miRNAs 12. The fact that p68 can unwind the let-7 miRNA duplex *in vitro*, suggests that p68 assists in the processing of pri-miRNAs by facilitating the loading of pri-miRNAs into the Drosha complex 13. Abnormal expression of miRNAs has been associated with the ADPKD phenotype 14. It has been found that miR-17 family and miR-21 are both upregulated in cystic kidneys and promote ADPKD progression in mouse models 15. Also, the proto-oncogene c-Myc transactivates miR-17~92, which in turn rewires cyst epithelial metabolism to enhance cyst proliferation. However, the upstream regulator for miRNA biogenesis and maturation in ADPKD is not known.

Although there is extensive evidence that has deciphered the central roles of p68 with respect to intracellular signaling networks, little is known about its expression and its role in the transcriptional regulation of PKD genes in ADPKD kidneys. Moreover, the cellular consequences of abnormal p68 expression are not yet completely understood. Such knowledge might provide invaluable insights into the molecular mechanisms regarding p68 in the context of cystogenesis. In this study, we found that p68 was upregulated in *Pkd1* mutant renal epithelial cells and tissues. We demonstrate that p68 cooperates with p53 to regulate the transcription of the *Pkd1* gene, and cooperates with Drosha to regulate the expression of PKD associated miRNAs which further affect *Pkd1* gene post-transcriptional regulation. We also show that p68 regulates the phosphorylation and activation of ERK, mTOR, and Rb signaling pathways in *Pkd1* mutant renal epithelial cells, and we show that the expression of p68 can be stimulated by oxidative stress and TGF-β1. Moreover, knockdown of p68 display a significantly lower lumen expansion and cyst formation in a 3D spheroids model of mouse collecting duct cells. Our results suggest that p68 is a key molecule involved in the regulation of the expression of the *Pkd1* gene and PKD associated miRNAs as well as the activation of PKD regulated signaling pathways, providing a rationale to develop new therapeutic strategies for ADPKD treatment once a p68 inhibitor becomes available.

## Materials and Methods

### Human samples

De-identified human ADPKD and normal human kidney formalin-fixed, paraffin-embedded sections were obtained from the Mayo Translational Polycystic Kidney Disease Center. Informed consent was obtained.

### Mouse kidney samples

*Pkd1^fl/fl^:Pkhd1-Cre* mice were generated by cross-breeding *Pkd1^fl/+^:Pkhd1-Cre* female mice with *Pkd1^fl/+^:Pkhd1-Cre* male mice. The kidneys were harvested and analyzed at postnatal day 21. Littermate controls were used in all animal experiments.

### Cell culture and reagents

Mouse inner-medullary collecting duct 3 (mIMCD3) cells and renal cortical tubular epithelia (RCTE) cells were maintained at 37 °C in 5% CO_2_ in DMEM (Invitrogen) supplemented with 10% FBS. *Pkd1* WT and *Pkd1*-null MEK cells, derived from collecting ducts and sorted by the collecting duct marker Dolichos Biflorus Agglutinin (DBA) from kidneys of WT and *Pkd1*-null mice, were maintained as previously described 16, 17. PH2 and PN24 cells (provided by S. Somlo through the George M. O'Brien Kidney Center, Yale University, New Haven, Connecticut, USA) were cultured as described previously 18.

The antibodies used for Western blot analysis included (a) anti-p68 (sc-166167), anti-p53 (sc-6423), anti-Fibronectin (sc-59826), anti-Transforming growth factor-beta 1 (TGF-β1) (sc-146), anti-CyclinD1 (sc-450) purchased from Santa Cruz Biotechnology Inc.; (b) anti-S6 (no. 2217), anti-Rb (no. 9309), anti-ERK (no. 4696) and anti-H2AX (no.2595) purchased from Cell Signaling Technology; (c) the phosphorylated antibodies for ERK-T202/Y204 (no. 9101), S6-S235/236 (no. 2211) and Rb-S780 (no. 9307) also purchased from Cell Signaling Technology; (d) anti-Drosha (ab12286) purchased from Abcam; (e) anti-PC1 (7e12) was provided by Dr. James P. Calvet's lab; (f) anti-γH2AX(Ser139) was purchased from Millipore; and (g) anti-actin antibody (A5316) was purchased from Sigma-Aldrich. The secondary antibodies, including donkey anti-rabbit IgG-horseradish peroxidase (sc-2313) and goat anti-mouse IgG-horseradish peroxidase (sc-2005) were purchased from Santa Cruz Biotechnology, Inc.

The antibodies used for Immunofluorescent staining included (a) anti-p68 antibody (sc-166167, 1:200), anti-PCNA antibody (sc-56, 1:200) were purchased from Santa Cruz Biotechnology Inc.; (b) anti-γH2AX(Ser139) was purchased from Millipore; (c) Alexa Fluor secondary antibody 555 or 488 were purchased from Thermo Fisher; (d) Lotus Tetragonolobus Lectin (LTL) (FL-1321) and Dolichos Biflorus Agglutinin (DBA) (FL-1031) were purchased from Vector Laboratories.

*mir*Vana® mmu-miR-182-5p inhibitor (MH13088) and negative control were purchased from Thermo Fisher. *mir*Vana® mmu-miR-182-5p miRNA mimic (MC13088) and negative control were purchased from Thermo Fisher.

### Small interfering RNA (siRNA) transfection

The RNA oligonucleotides that specifically target mouse p68 and mouse p53 were purchased from Santa Cruz Biotechnology, Inc. The RNA oligonucleotides were transfected with Dharma-FECT siRNA transfection reagent (Dharmacon). 24 h or 48 h after transfection, cells were harvested and analyzed by Western blotting.

### RNA extraction and quantitative reverse transcription-polymerase chain reaction (qRT-PCR)

Total RNA was extracted using the RNeasy Plus Mini Kit (QIAGEN). Total RNA (1 μg) was used for RT reactions in a 20 μl reaction to synthesize cDNA using an iScript cDNA Synthesis Kit (Bio-Rad). RNA expression profiles were analyzed by real-time PCR using iTaq SYBR Green Supermix with ROX (Bio-Rad) in an iCycler iQ Real-Time PCR Detection System. The complete reactions were subjected to the following program of thermal cycling: 40 cycles of 10 s at 95 °C and 20 s at 60 °C. A melting curve was run after the PCR cycles followed by a cooling step. Each sample was run in triplicate in each experiment, and each experiment was repeated 3 times. Expression levels of target genes were normalized to the expression level of actin. All primers used are listed in Table 1.

### Quantification of miRNA amplification

Total RNA was extracted using Trizol reagent, and reverse transcription was carried out with MicroRNA first-strand synthesis and miRNA quantitation kits (Takara, Japan) according to the manufacturer's protocol. Analyses were carried out using the SensiFAST SYBR No-ROX Kit (Bioline, USA). qRT-PCR was conducted with 40 cycles of 10 min incubation at 95 °C, 15 s incubation at 95 °C, and 1 min incubation at 60 °C. All primers were ordered from Integrated DNA Technologies. The values for gene expression were calculated with the 2^-ΔΔCt^ method.

### Protein extraction and Western blot analysis

Cell pellets were collected and resuspended in lysis buffer (20 mM Tris-HCl, pH 7.4, 150 mM NaCl, 10% glycerol, 1% Triton X-100, 1 mM Na_3_VO_4_, 25 mM β-glycerolphosphate, 0.1 mM PMSF, Roche complete protease inhibitor set, and Sigma-Aldrich phosphatase inhibitor set). The resuspended cell pellet was vortexed for 20 s and then incubated on ice for 30 min and centrifuged at 20,000 g for 30 min. The supernatants were collected for Western blot analysis or immunoprecipitation.

For immunoprecipitation, anti-p68, anti-Drosha or anti-53 antibodies and their isotype control antibodies were coupled to protein A agarose beads (Pierce) in PBS containing 5 mg/mL bovine serum albumin (Sigma-Aldrich) for 6 h at 4 °C on a rotating platform. The cell lysates were then incubated with the beads coupled with antibodies overnight at 4 °C. The next day, beads were washed with lysis buffer containing an additional 300 mM NaCl, and the immune complexes were eluted off the beads using loading buffer with boiling for 5 min and then subjected to Western blot analysis.

### ChIP assay

ChIP assays were performed according to the protocol described previously 1. Chromatin DNA was subjected to immunoprecipitation with anti-p68, anti-p53, anti-RNA polymerase II antibody, or normal rabbit IgG, and then washed, after which the DNA-protein crosslinks were reversed. The recovered DNA was analyzed by PCR for the binding of p68 or p53 at the *Pkd1* promoter.

### Tissue immunofluorescence staining

After antigen retrieval, tissue sections were incubated with primary antibody overnight, and then incubated with Alexa Fluor secondary antibody or LTL, or DBA and mounted in Prolong Gold Antifade reagent with DAPI (Invitrogen). Images were analyzed using a Nikon Eclipse 80i microscope.

### DNA damage response (DDR) foci count

Confocal images were collected at the same conditions for control and treatment groups 19. Mean γH2AX fluorescence intensity per nucleus was automatically measured with NIH image J (http://rsb.info.nih.gov/ij/). Approximately 150 events per condition were scored.

### MTT assays

Cell proliferation was measured using an MTT-based kit (Sigma-Aldrich) according to the manufacturer's instructions.

### 3D spheroid model for mIMCD3 cells and immunofluorescence

Mouse IMCD3 cells were forward transfected with p68-siRNA or negative control (NC) -siRNA and then were seeded in Matrigel 20. In brief, mIMCD3 cells were treated with trypsin for 3-5 min and resuspended in prewarmed (37 °C) medium in a concentration of 100,000 cells per milliliter. Next, 100 μl of cell suspension was gently mixed with 100 μl of growth factor-depleted Matrigel (BD Biosciences) in a 1.5 ml microcentrifuge tube. Then the cell-Matrigel mixture was transferred to each well of a 24 well plate with coverslip. After polymerization for 30 min at 37 °C, warmed complete culture medium was dripped over the matrix until the well was just covered. The cells were cultured at 37 °C with 5% CO_2_. In general, the cells formed spheroids with cleared lumens in 2 to 3 days. To prepare spheroids for qRT-PCR, Western blot or immunofluorescence assay, we dissolved matrigel and collected 3D spheroids followed the protocol 20. Briefly, the 3D culture media were replaced with chilled cell recovery solution (BD Biosciences, 354253), then the Matrigel was carefully broken up with no damage on the 3D cultures by pipette and incubation until the Matrigel was fully depolymerized and 3D cultures were floating free from Matrigel. The cell recovery solution was removed by centrifugation and the cultures were washed with cold PBS. The samples are ready for protein extraction or RNA extraction. For immunofluorescence staining, 4% Paraformaldehyde Fix Solution (PFA) was added to dissolve the Matrigel. Then wash with Dulbecco's PBS (DPBS) to remove the Matrigel and collect the spheroids to perform the immunofluorescence procedure. Immunofluorescence was performed as described and imaged using a Zeiss LSM 780 inverted confocal microscope system.

### Statistical analysis

All values were presented as the mean ± SD and analyzed with SPSS, version 17.0. Differences in mean values were tested using Student's t test or one-way analysis of variance. Differences with P values less than 0.05 were considered statistically significant.

### Study approval

These animal experiments were conducted under the following IACUC protocol: A00003756-18. They were reviewed and approved by the IACUC of the Mayo Clinic, in accordance with the National Institutes of Health, United States Department of Agriculture, and the Association for Assessment and Accreditation of Laboratory Animal Care guidelines.

## Results

### p68 regulates *Pkd1* gene transcription via promoter binding

**Figure 1:**
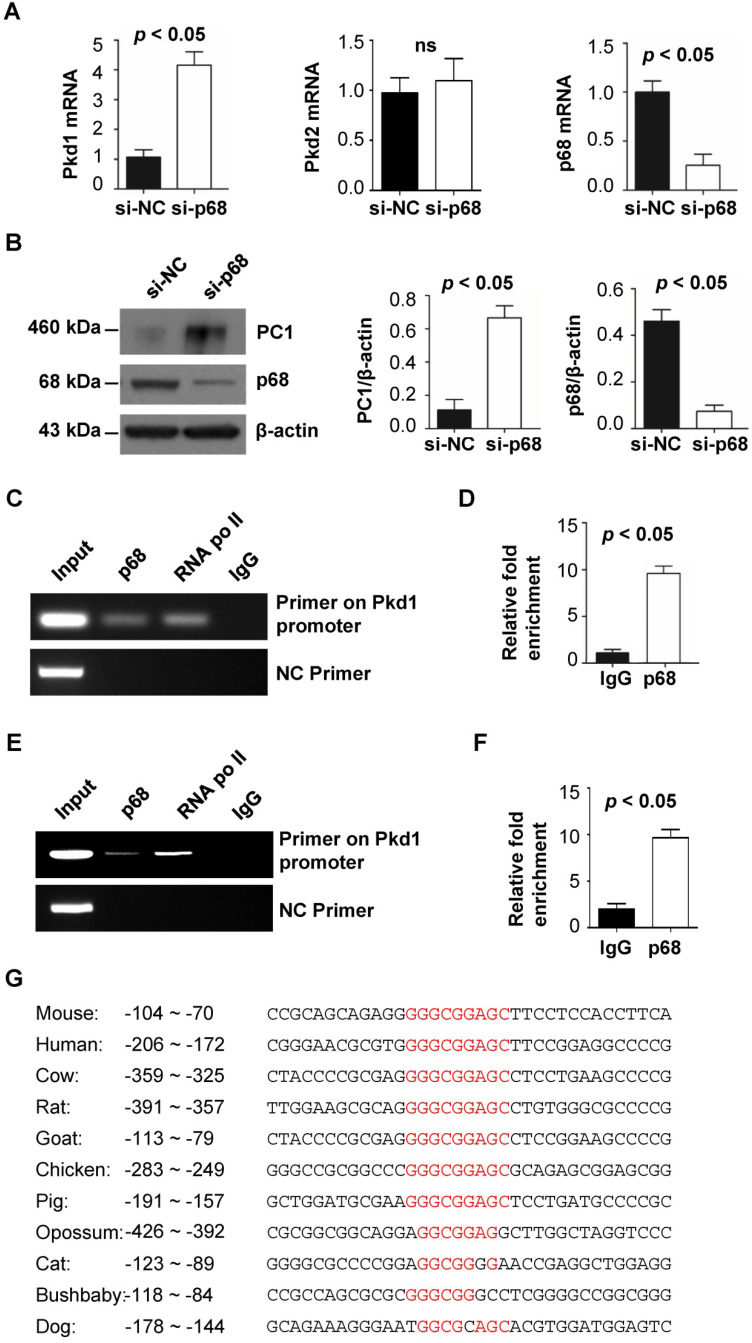
** p68 represses *Pkd1* expression by binding to the *Pkd1* promoter.** (**A**) The expression levels of *Pkd1*, *Pkd2* and p68 were analyzed by qRT-PCR in mIMCD3 cells transfected with p68-siRNA or Negative Control (NC)-siRNA for 24 h. (**B**) The expression levels of p68 and PC1 were analyzed by Western blot in mIMCD3 cells transfected with p68-siRNA or NC-siRNA for 24 h. (**C**, **D**) ChIP and ChIP-qPCR assay was performed with anti-p68 antibody or normal mouse IgG in mIMCD3 cells. Anti-RNA polymerase II antibody was used as a positive control. Negative control (NC) primers were located about 2000 bp upstream of the transcription start site (TSS) of mouse *Pkd1* gene. (**E**, **F**) ChIP and ChIP-qPCR assay was performed with anti-p68 antibody or normal mouse IgG in RCTE cells. Anti-RNA polymerase II antibody was used as a positive control. Negative control (NC) primers were also located about 2000 bp upstream of the transcription start site (TSS) of human *PKD1* gene. (**G**) Alignment of *Pkd1* promoter among species identified a consensus sequence, GGGCGGAGC, which may be a p68 binding motif.

**Figure 2:**
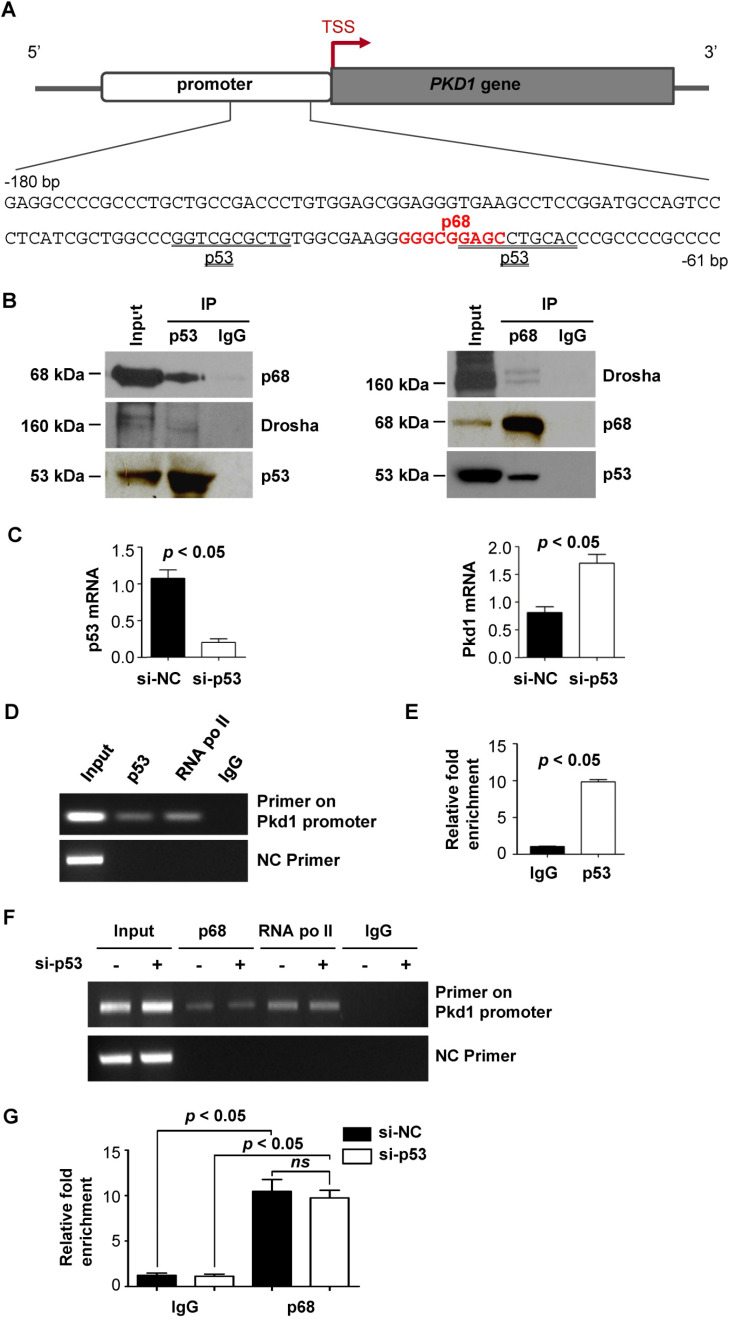
** p68, p53 and Drosha form a ternary complex to regulate *Pkd1* transcription.** (**A**) Sequence (-180 to -61 bp) of the proximal promoter of the human *PKD1* gene includes a p68 (red) and a p53 (underlined) motif upstream of the TSS. (**B**) The interactions between p53 and p68 and between p53 and Drosha in mIMCD3 cells were detected with anti-p53 antibody followed by blotting with Drosha antibody and p68 antibody, respectively (*left panel*). The interactions between p68 and Drosha and between p68 and p53 in mIMCD3 cells were detected with anti-p68 antibody followed by blotting with Drosha and p53 respectively (*right panel*). IgG was used as a negative control. (**C**) The mRNA levels for p53 and *Pkd1* were analyzed by qRT-PCR in mIMCD3 cells transfected with p53-siRNA or NC-siRNA for 24 h. (**D, E**) ChIP and ChIP-qPCR analysis was performed with anti-p53 antibody or normal mouse IgG in mIMCD3 cells. Anti-RNA polymerase II antibody was used as a positive control. Negative control (NC) primers were located about 2000 bp upstream of the transcription start site (TSS) of mouse *Pkd1* gene. (**F, G**) ChIP and ChIP-qPCR analysis was performed with anti-p68 antibody or normal mouse IgG in mIMCD3 cells transfected with p53 siRNA and control siRNA (si-NC). Negative control (NC) primers were located about 2000 bp upstream of the transcription start site (TSS) of mouse *Pkd1* gene.

Both *Pkd1* haploinsufficiency and overexpression can result in renal cyst formation in mouse models, suggesting that the expression of the human *PKD1* gene should be tightly controlled. Because p68 is known to be important in transcriptional regulation, we investigated its role in regulating the expression of the *Pkd1* gene and PC1 protein. We found that knockdown of p68 with siRNA increased *Pkd1* mRNA and PC1 protein levels in mouse inner-medullary collecting duct (mIMCD3) cells compared to cells transfected with control siRNA, as examined by quantitative RT-PCR (qRT-PCR) (Figure 1A) and Western blot (Figure 1B) analysis. But knockdown of p68 did not result in the change of *Pkd2* mRNA in mIMCD3 cells compared to that in control siRNA transfected cells as examined with qRT-PCR analysis (Figure 1A). These results suggested that p68 has no effect on the transcription of *Pkd2* gene in renal epithelial cells. To support the idea that p68 directly regulates the expression of *Pkd1* in renal epithelial cells, we performed chromatin immunoprecipitation (ChIP) assays. We found that p68 was recruited to the promoter of the *Pkd1* gene within the -65 to -209 bp region upstream of the transcription start site (+1) in mIMCD3 cells (Figure 1C). The enrichment of p68 on the *Pkd1* promoter is about 9.2-fold more than the binding of IgG on the promoter of *Pkd1* gene (Figure 1D). We also further confirmed that p68 was recruited to the promoter of the *PKD1* gene within the -114 to -286 bp region upstream of the transcription start site (+1) in RCTE cells (Figure 1E), and the enrichment of p68 on the *PKD1* promoter in these cells is about 8.9 -fold more than the binding of IgG on the promoter of *PKD1* gene (Figure 1F). Analysis for putative cis-acting elements revealed that there are no typical TATA and CAAT boxes within the 5'-flanking region of *Pkd1* promoter. However, with alignment of the sequences of *Pkd1* promoter among different species, we identified one conserved p68 binding motif, GGGCGGAGC in *Pkd1* promoter (Figure 1G, Figure 2A). Our results suggest that p68 plays a role in repressing *Pkd1* gene transcription.

### p68 interacts with p53 and works together with p53 to regulate the transcription of *Pkd1*

*PKD1* proximal promoter region contains an atypical p53 binding motif 21, which can mediate p53-dependent transcriptional repression (Figure 2A,* underline*). Interestingly, we found that the p68 binding motif partially overlapped with the p53 binding motif in *Pkd1* promoter (Figure 2A). It has been reported that p68 is an established co-factor of p53 in regulating gene transcription 9, we investigated whether p68 works together with p53 to regulate transcription of the *Pkd1* gene. We found that p68 interacts with p53 using an anti-p53 antibody to pull down p68 (Figure 2B, *left panel*) and an anti-p68 antibody to pull down p53 (Figure 2B, *right panel*) from mIMCD3 cells. We further found that knockdown of p53 with siRNA increased the mRNA levels of *Pkd1* in these cells (Figure 2C). Our ChIP assay found p53 bound to the promoter of *Pkd1* gene within the same -209 to -65 bp region upstream of transcription start site (+1) as did by p68, to negatively regulate the transcription of *Pkd1* gene (Figure 2D and 2E). To investigate whether the recruitment of p68 to the *Pkd1* promoter depends on p53, we knocked down p53 with siRNA in mIMCD3. Our ChIP and ChIP qPCR assays indicated that p68 was recruited to the *Pkd1* promoter both in p53 silencing cells and control siRNA transfected cells (Figure 2F and 2G), supporting that the recruitment of p68 to the promoter of *Pkd1* is independent of p53. Our results suggest that both p68 and p53 bound on the promoter of *Pkd1* gene to repress its transcription.

### DNA damage induced the downregulation of *Pkd1* in a p68-dependent manner

**Figure 3:**
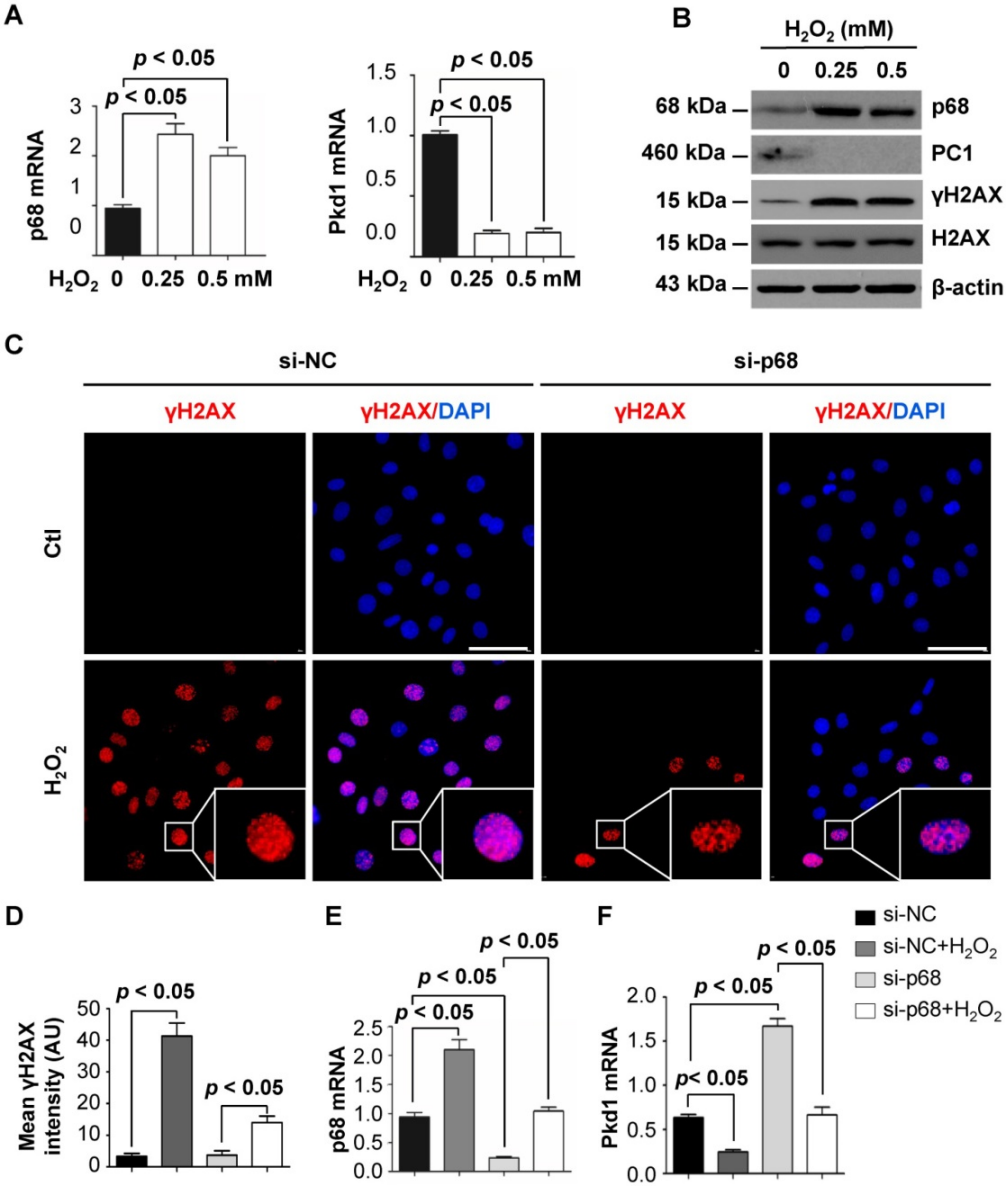
** p68 decreases *Pkd1* gene expression in response to oxidative DNA damage.** (**A**) qRT-PCR analysis was performed to detect mRNA levels of p68 and *Pkd1* in mIMCD3 cells treated with H_2_O_2_ at the indicated concentrations for 6h. (**B**) Western blot analysis was performed to detect protein levels of p68, PC1, γH2AX and H2AX in mIMCD3 cells treated with H_2_O_2_ at the indicated concentrations for 6h. (**C**) Immunofluorescent staining of γH2AX in mIMCD3 cells transfected with p68 siRNA or control siRNA following the treatment with or without H_2_O_2_ (0.5 mM) for 6 h. Scale bar, 50 µm. (**D**) Quantification of γH2AX staining intensity per nucleus in mIMCD3 cells transfected with p68 siRNA or control siRNA following the treatment with or without H_2_O_2_ for 6 h (100 cells scored per condition in three independent experiments). (**E, F**) qRT-PCR analysis was performed to detect mRNA levels of p68 and *Pkd1* in mIMCD3 cells transfected with p68 siRNA or control siRNA and then treated with or without H_2_O_2_ (0.5 mM) for 6h.

Stress stimuli and DNA damage have been found to play roles in inhibiting *PKD1* gene expression, possibly causing haploinsufficiency and cyst formation 22. However, the molecular mechanisms involved in this process are unclear. As DNA damage can be induced by high levels of H_2_O_2_ 23 , we investigated the effects of H_2_O_2_ treatment on p68 and *Pkd1* gene expression. We found that H_2_O_2_ treatment increased the mRNA levels of p68 but decreased the mRNA level of *Pkd1* (Figure 3A) as examined in mIMCD3 cells by qRT-PCR analysis. Consistent with these mRNA results, we also found that H_2_O_2_ treatment increased the protein levels of p68 but decreased the protein level of PC1. In addition, we found that treatment with H_2_O_2_ increased the levels of phosphorylated H2AX (γH2AX), a marker for DNA damage signaling activation (Figure 3B). Our immunofluorescence staining with γH2AX antibody confirmed higher intensities of γH2AX expression per nucleus in the H_2_O_2_ treated mIMCD3 cells. However, knockdown of p68 significantly decreased the γH2AX positive nuclei compared to those in the control cells (Figure 3C and 3D). To further assess whether p68 was involved in this DNA damage-induced reduction in *Pkd1* expression, we knocked down p68 with siRNA and then treated the cells with H_2_O_2_. With this treatment, we found that *Pkd1* reduction was restored following p68 knockdown (Figure 3E and 3F). These results suggest that downregulation of *Pkd1* transcription by DNA damage (H_2_O_2_ treatment) is dependent on p68 induction.

### p68 regulates miR182-5p processing to induce cleavage of *Pkd1* mRNA

**Figure 4:**
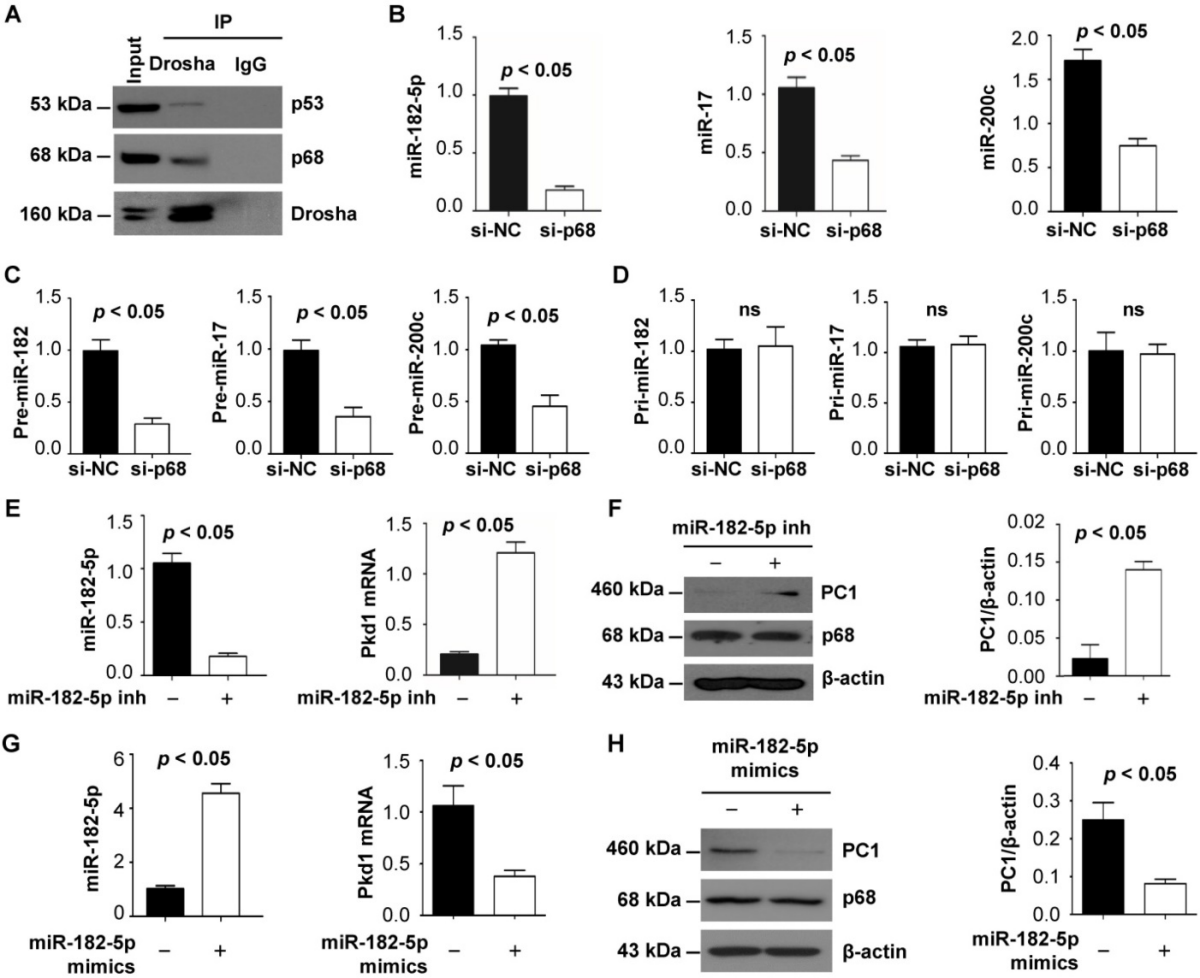
** p68 regulates *Pkd1* expression by miR-182-5p mediated post-transcriptional repression.** (**A**) The interaction between Drosha and p53 (top panel) and between Drosha and p68 (middle panel) was detected in mIMCD3 cells with anti-Drosha antibody followed by blotting with p68 and p53. IgG was used as a negative control. (**B** to** D**) The expression of miR-182-5p, miR-17 and miR-200c, pre-miR-182-5p, pre-miR-17 and pre-miR-200c, and pri-miR-182-5p, pri-miR-17 and pri-miR-200c in mIMCD3 cells transfected with p68 or control siRNA examined by qRT-PCR analysis. (**E**, **F**) The levels of *Pkd1* mRNA and PC1 protein in mIMCD3 cells treated with a miR-182-5p inhibitor examined by qRT-PCR and Western blot analysis. (**G, H**) The levels of *Pkd1* mRNA and protein were examined by qRT-PCR (**G**) and Western blot analysis (**H**) in mIMCD3 cells treated with a miR-182-5p mimics.

Identification of p68 as a component of the Drosha complex highlights its involvement in miRNA biogenesis 11 and suggests a possible connection between p68, miRNAs and ADPKD. We first confirmed the interaction of p68 and Drosha by co-IP analysis in mIMCD3 cells and found that both p68 and p53 could pull down Drosha (Figure 2B) and that Drosha could also pull down p68 and p53 (Figure 4A), suggesting that p68 might be an upstream regulator of miRNA biogenesis by formation of a ternary complex with Drosha (Figure 2B, Figure 4A). Bioinformatic analysis predicted that miR-17, miR-200c and miR-182 might bind to 3'-UTR sites of *Pkd1* mRNA. We found that knockdown of p68 decreased the levels of miR-17, miR-200c and miR-182-5p in mIMCD3 cells (Figure 4B) and their precursors, precursor (pre) -miR-17, pre-miR-200c and pre-miR-182 (Figure 4C), but not pri-miR-17, pri-miR-200c and pri-miR-182 (Figure 4D). These data support a role for p68 in modulating the maturation of miR-17, miR-200c and miR-182-5p. It has been reported that miR-17 and miR-200 are involved in regulating the transcription of the *Pkd1* gene 29. However, the role of miR-182-5p in regulating *Pkd1* transcription is still unknown. To examine this possibility, we treated mIMCD3 cells with miR-182-5p inhibitor and found that inhibition of miR-182-5p decreased the expression of miR-182-5p (Figure 4E) and increased *Pkd1* mRNA and protein levels in mIMCD3 cells (Figure 4E and 4F). Furthermore, to investigate the role of miR-182-5p overexpression in the regulation of PC1 expression, we treated *Pkd1* wild type mouse embryonic kidney (MEK) cells with miR-182-5p mimics to increase the expression of miR-182-5p (Figure 4G) in these cells. We found that this treatment increased *Pkd1* mRNA and protein levels in *Pkd1* WT MEK cells (Figure 4G and 4H). These results suggest that p68 functions as a regulator of the expression and maturation of PKD associated miRNAs, including miR-17, miR-200c and miR-182-5p, and represses the expression of the *Pkd1* gene via these miRNAs during cyst progression.

### The expression of p68 is upregulated in *Pkd1* mutant renal epithelial cells and tissues

**Figure 5:**
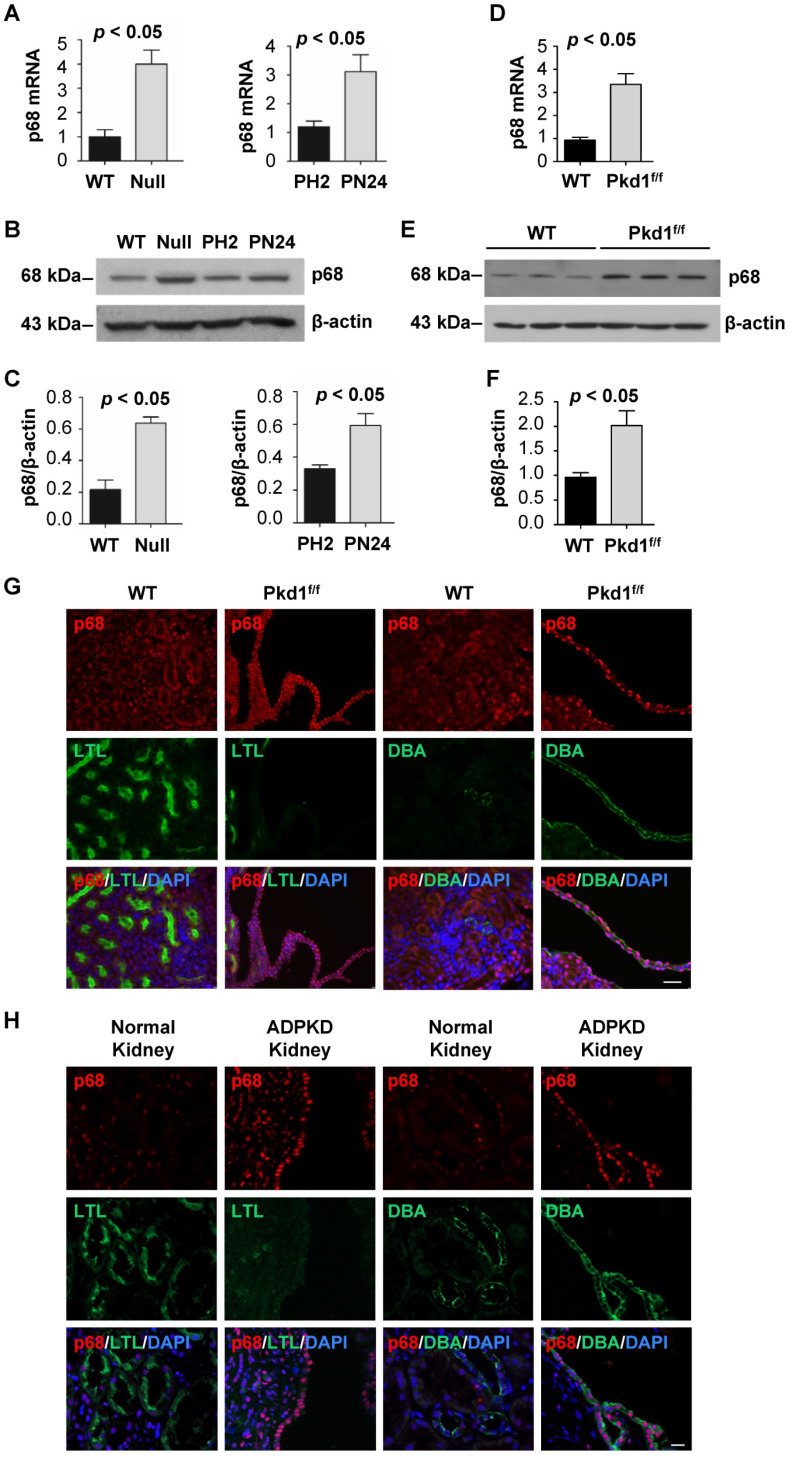
** The expression of p68 is increased in *Pkd1* mutant renal epithelial cells and tissues.** (**A** to** C**) The expression of p68 in Pkd1 (WT) and Pkd1 mutant (Null) MEK cells, and in postnatal Pkd1 heterozygous PH2 (PH2) cells and Pkd1 homozygous PN24 (PN24) cells was examined by qRT-PCR (**A**) and Western blot (**B**) analysis; The relative p68 protein level was quantified from 3 independent immunoblots compared to actin (**C**). (**D** to** F**) The expression of p68 in postnatal day 21 kidneys from *Pkd1^+/+^: Pkhd1-Cre* (WT) and *Pkd1^fl/fl^: Pkhd1-Cre* (*Pkd1^fl/fl^*) mice was examined by qRT-PCR (**D**) and Western blot (**E**, **F**) analysis. (**G**) The expression of p68 was examined in postnatal day 21 kidneys from *Pkd1^+/+^: Pkhd1-Cre* (WT) and *Pkd1^fl/fl^: Pkhd1-Cre* (*Pkd1^fl/fl^*) mice with anti-p68 antibody and co-stained with LTL or DBA. (**H**) The expression of p68 was examined in kidneys from ADPKD patients and normal individuals with anti-p68 antibody and co-stained with LTL or DBA, which indicated that p68 expression was increased in cyst-lining epithelia in ADPKD kidneys but not in normal human kidneys. Scale bars, 50 µm.

To further understand the role of p68 in ADPKD, we investigated the expression of p68 in *Pkd1* mutant renal epithelial cells and tissues. We found that p68 was upregulated in *Pkd1* null MEK cells and postnatal *Pkd1* homozygous PN24 cells compared to *Pkd1* WT MEK cells and *Pkd1* heterozygous PH2 cells as examined by qRT-PCR (Figure 5A) and Western blot analysis (Figure 5B and 5C). In addition, we found that the mRNA and protein levels of p68 were increased in *Pkd1* conditional knockout mouse kidneys (Figure 5D, 5E and 5F). Our immunostaining analysis further indicated that p68 was elevated in DBA, a collecting duct marker, positive cyst lining epithelia in *Pkd1* conditional knockout mouse kidneys (Figure 5G) and human ADPKD kidneys but not in wild type (WT) and normal human kidneys (Figure 5H). The absence of p68 in LTL, a proximal tubule marker, positive tubules suggests that the increased expression of p68 is due to mutation of *Pkd1* in collecting ducts in mouse and human kidneys.

### p68 promotes renal cell proliferation by activating ERK, mTOR, and Rb pathways

**Figure 6:**
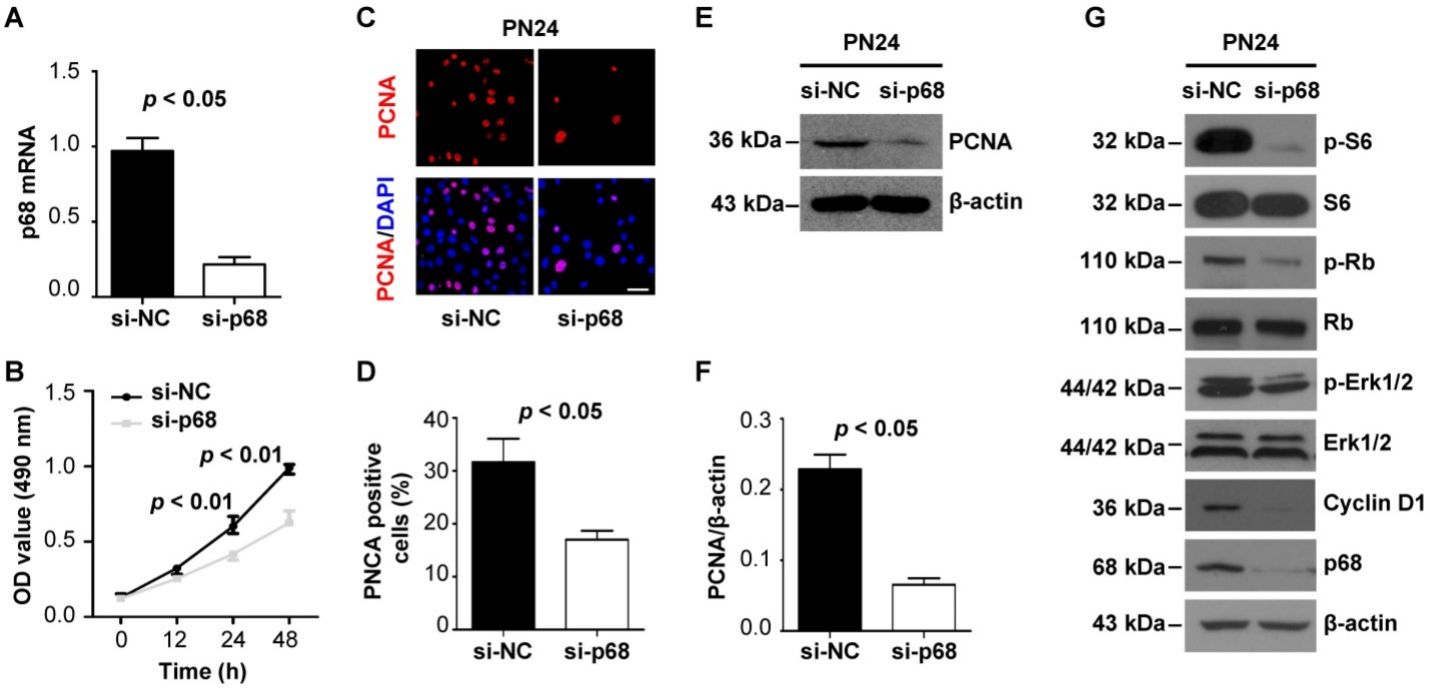
** p68 promotes renal epithelial cell proliferation by activating ERK, mTOR, and Rb pathways.** (**A**) The expression of p68 was examined with qRT-PCR analysis in PN24 cells transfected with p68 siRNA or control siRNA for 48 h. (**B**) Cell growth of PN24 cells transfected with p68 siRNA or control siRNA was determined by MTT assay. (**C** to** F**) The expression of PCNA in *Pkd1* mutant PN24 cells transfected with p68 siRNA or control siRNA for 48 h was examined with PCNA staining (**C, D**) and Western blot (**E, F**). Scale bars, 50 µm. (**G**) Western blot analysis of the expression of Cyclin D1 and the phosphorylation of ERK, S6 and Rb as well as the total levels of these proteins in *Pkd1* homozygous PN24 transfected with p68 siRNA or control siRNA for 48 h.

As p68 has been reported to participate in cancer development and progression by functioning in several key cellular activities of cancer cells, such as cancer cell proliferation 24, 25, we examined p68-regulated cell proliferation in *Pkd1* mutant renal epithelial cells. We found that knockdown of p68 with siRNA significantly decreased the growth of *Pkd1* homozygous PN24 cells and Pkd1 null MEK cells compared to cells transfected with control siRNA as examined by MTT assay (Figure 6A and 6B, Figure S1A and S1B). We also found that the staining of proliferating cell nuclear antigen (PCNA) (Figure 6C and 6D, Figure S1C and S1D) and the expression of PCNA were decreased in p68 knockdown cells compared to those in the control cells (Figure 6E and 6F, Figure S1E and S1F). In addition, we found that knockdown of p68 in *Pkd1* homozygous PN24 cells and Pkd1 null MEK cells decreased the expression of Cyclin D1 and the phosphorylation of ERK, S6, and Rb, factors previously shown to be involved in regulating cystic epithelial cell proliferation and cyst growth 1 (Figure 6G and Figure S1G). These results suggest that upregulation p68 in cyst lining epithelial cells plays a role in regulating cyst growth in ADPKD.

### p68 promotes renal fibrosis via the TGF- β1 signaling pathway

**Figure 7:**
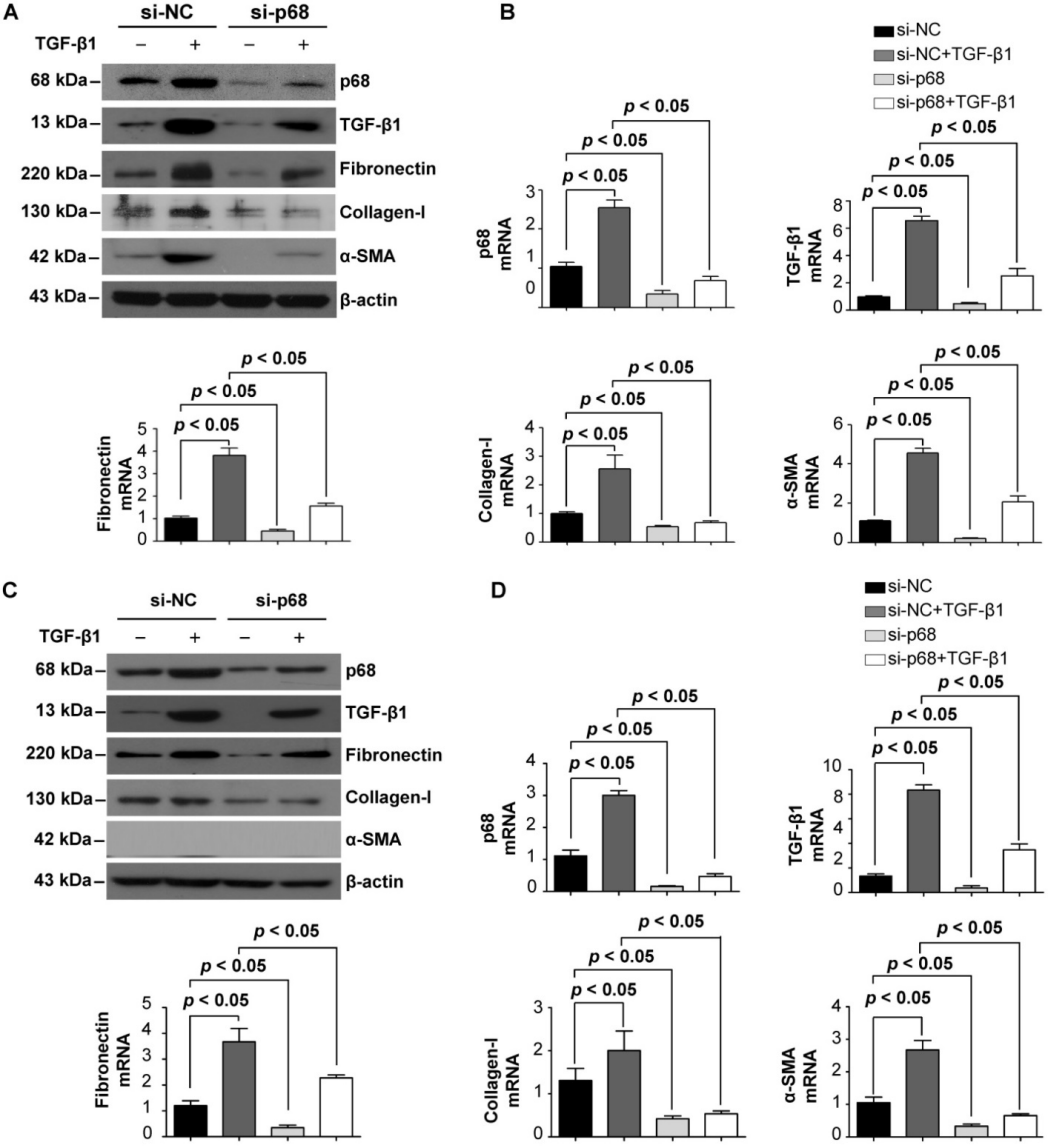
** TGF-β1 induces the expression of p68 and knockdown of p68 reverses TGF-β1 induced upregulation of fibrotic markers in cystic renal epithelial cells.** (**A, B**) The expression of p68, fibronectin, α-SMA, collagen-1 and TGF-β1 was examined with Western blot (**A**) and qRT-PCR (**B**) analysis in *Pkd1* homozygous PN24 cells transfected with p68 siRNA or control siRNA for 24 h and then treated with or without TGF-β1 (2 µg/ml) for 24 h. (**C, D**) The expression of p68, fibronectin, α-SMA, collagen-1 and TGF-β1 was examined by Western blot (**C**) and qRT-PCR (**D**) analysis in *Pkd1* null MEK cells transfected with p68 siRNA or control siRNA for 24 h and then treated with or without TGF-β1 (2 µg/ml) for 24 h.

Renal cyst progression results in an alteration in extracellular matrix (ECM) secretion and renal fibrosis 26. To investigate the role of p68 in renal fibrosis in ADPKD, we examined whether p68 regulates the expression of key fibrosis markers in cystic renal epithelial cells. We found that treatment with TGF-β1 induced the upregulation of mRNA and protein levels of p68 in *Pkd1* homozygous PN24 cells and Pkd1 null MEK cells. TGF-β1 treatment also increased the mRNA and protein levels of itself as well as fibronectin, α-smooth muscle actin (α-SMA) and collagen-1 (Figure 7A to 7D). However, knockdown of p68 decreased the mRNA and proteins levels of TGF-β1, fibronectin, α-SMA and collagen-1, and inhibited TGF-β1 induced upregulation of the fibrotic makers (Figure 7A to 7D). These results suggest that TGF-β1 regulates the expression of fibrotic markers via p68 in *Pkd1* mutant renal epithelial cells and that upregulation of p68 may contribute to renal fibrosis in ADPKD.

### p68 promotes cyst formation in mIMCD3 cell 3D-cultures

**Figure 8:**
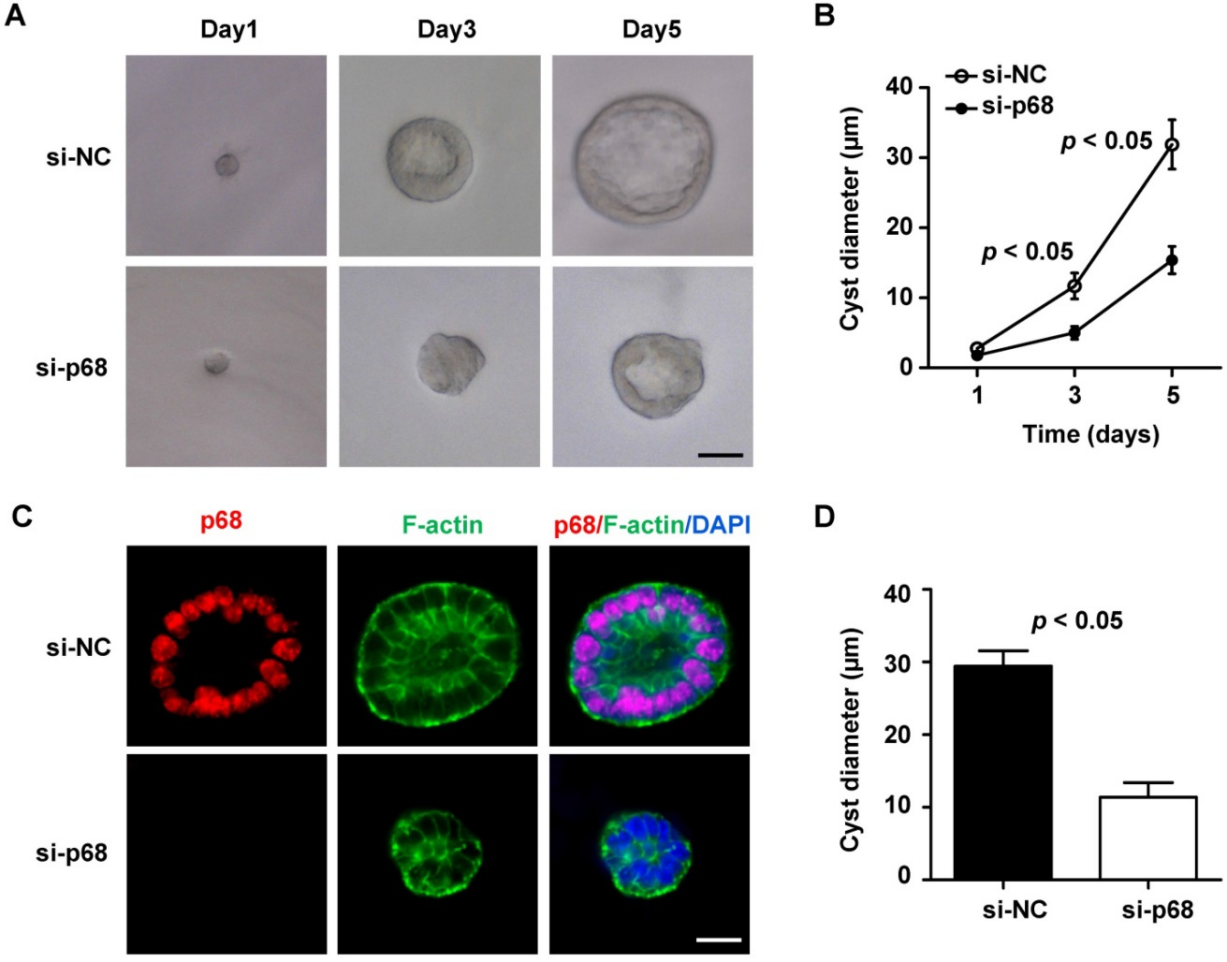
** Knockdown of p68 delays spheroid (cyst) growth in mIMCD3 cell 3D cultures.** (**A**) Light micrographs of cysts formed in Matrigel with mIMCD3 cells transfected NC-siRNA or p68-siRNA at the days 1, 3 and 5. Each series of photographs showed the same cyst on successive days in 3D cultures. Scale bars, 10 µm. (**B**) Cyst diameters of 3D cultures of mIMCD3 cells transfected with NC-siRNA or p68-siRNA on day 5. A total of 50 spheroids for each group from three different experiments were evaluated. (**C**) Spheroids were immunofluorescence-stained with antibodies against p68 and FITC-conjugated phalloidin for F-actin as well as DAPI. Spheroid morphology from mIMCD3 cells transfected with NC-siRNA or p68-siRNA (on day 5) judged by focusing to the equator plane of each spheroid using the z-axis of a confocal microscope (Leica). A total of 50 spheroids for each group from three different experiments were evaluated; scale bar, 10 µm. (**D**) The cyst diameters (µm) for NC-siRNA or p68 knockdown are plotted in a bar diagram. The area bounded by the F-actin of the cells is determined at the level of the largest lumen diameter of each cyst. Cysts that did not develop an open lumen were assigned an area of 0.

Given that p68 is involved in the regulation of *Pkd1* gene expression and signaling pathways associated with cystic renal epithelial cell proliferation and renal fibrosis, we are wondering whether p68 plays a critical role in renal cyst formation. Due to the lack of an available p68 knockout mouse model, we tested this possibility with a well-developed three-dimensional (3D) cell culture model that uses mIMCD3 cells to generate epithelial spheroids 27. We found that when mIMCD3 cell were cultured in Matrigel, a small ball of cells without a lumen was initially formed on day 1, and then the lumen-containing cysts were formed on day 3, which were progressively expanded up to day 5. To investigate the involvement of p68 in cyst formation in 3D cultures, we knocked down p68 in mIMCD3 cells with siRNA. First, we found that the expression of p68 protein and mRNA was decreased but the levels of PC1 and *Pkd1* mRNA was increased in 3D spheroids of mIMCD3 cells transfected with p68 siRNA compared to those in cells transfected with control siRNA (Figure S2). Next, we found that silence of p68 delayed cyst-like cell cluster formation, indicating by less discernible lumens, compared to those in the control siRNA 3D cultures (Figure 8A). The effect of silence of p68 on the lumen formation and expansion was similar as the observation of knockdown of grainyhead-like homolog 2 in 3D IMCD cell cultures 28. We further found that knockdown of p68 significantly decreased the diameters of cysts formed in 3D cultures at day 3 (5.5 ± 1.0 µm) and day 5 (17.2± 1.7 µm) compared to those in control siRNA 3D cultures at day 3 (14.6 ± 1.58 µm) and day 5 (32.8± 3.0 µm), respectively (Figure 8B). By staining the spheroids with p68, FITC-phalloidin for F-actin and DAPI (for nuclear staining) to visualize spheroid architectures under an inverted confocal microscopy, we found that cyst lumens which were surrounded with polarized epithelial cells as outlined by F-actin to the mark the membrane were always formed on mIMCD3 cell (control siRNA) 3D cultures, whereas the lumens failed to be expanded as seen with an undetectable or markedly diminished luminal space in the 3D cultures with p68 knockdown cells (Figure 8C). Our quantification of the defects in the expansion of lumens bounded by the F-actin-marked membrane at the center and the maximal lumen diameter of each cyst revealed profound differences in lumen size between control siRNA and p68 knockdown cells (Figure 8D). Together, these results indicated that knockdown of p68 delayed the lumen formation and expansion in 3D cultures.

## Discussion

**Figure 9:**
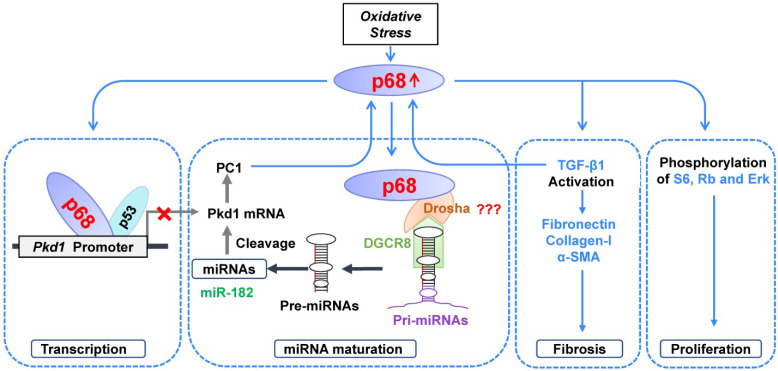
** Working model**. In ADPKD, DNA damage and oxidative stress induce upregulation of p68, which 1) negatively regulates *Pkd1* gene transcription by binding to the *Pkd1* promoter together with p53, 2) positively regulates the expression and maturation of PKD-associated miRNAs leading to posttranscriptional cleavage and loss of *Pkd1* mRNA, 3) positively regulates the expression of fibrotic markers, and 4) stimulates the phosphorylation and activation of PKD-associated signaling pathways, resulting in an increase of cystic renal epithelial cell proliferation and fibrosis in ADPKD kidneys.

Various studies have suggested that the dosage of polycystins below or above a critical threshold results in renal cyst formation 29. However, the molecular mechanisms for the transcriptional and posttranscriptional regulation of the *PKD1* gene under normal, cystic and stress stimuli conditions remain largely unknown. In this study, we show that a DEAD box containing RNA helicase, p68, represses *Pkd1* gene expression via transcriptional and posttranscriptional mechanisms in renal epithelial cells, by showing 1) that p68 binds the *Pkd1* promoter in association with p53 to repress transcription; and 2) that p68 promotes the expression and maturation of miR-182 to increase the cleavage and inactivation of *Pkd1* mRNA, through a process involving a complex between p68 and Drosha (Figure 9). We also found that treatment of cells with H_2_O_2_ increased mRNA and protein levels of p68 which then caused decreased *Pkd1* mRNA and PC1 protein levels. We also found that p68 was upregulated in *Pkd1* mutant renal epithelial cells and cyst lining epithelial cells in *Pkd1* knockout mouse kidneys and ADPKD patient kidneys. As in the mouse, we would expect that upregulation of p68 in cystic renal epithelial cells in ADPKD kidneys should decrease the transcription of the *PKD1* gene. In this study we present a negative feedback loop between p68 and *Pkd1*, in that p68 as an upstream regulator of *Pkd1*, and downregulation of *Pkd1* resulted in the upregulation of p68, which was proved in *Pkd1* knockout mouse kidneys (Figure 5D-G). We also showed that knockdown of p68 in *Pkd1* mutant renal epithelial cells decreased the phosphorylation and activation of PKD associated signaling targets, including ERK, S6, and Rb, and the expression of Cyclin D1, as well as the expression of fibrotic markers, including fibronectin, collagen-1, α-SMA and TGF-β1. Due to the lack of an available p68 knockout mouse model, we could not evaluate the role of p68 on renal cyst growth *in vivo*. However, we found that silence of p68 delayed cysts formation and growth in a highly acceptable mouse IMCD 3D spheroid model. Our results suggest that p68 plays an important role in regulating the transcription of the *PKD1* gene in ADPKD, the expression and maturation of miRNAs, the expression of fibrotic markers and the activation of PKD associated signaling pathways to promote cyst growth and renal fibrosis in ADPKD. Targeting p68 should be a novel therapeutic strategy for ADPKD treatment once an inhibitor of p68 becomes available.

p68 is a prototypic multifunctional protein involved in regulating a diverse range of cellular processes, including positively and negatively regulating gene transcription and pre-mRNA, rRNA and miRNA processing 30. Abnormal expression of p68 has been detected in many cancers, such as colon cancer, breast cancer, leukemia, and so on. However, the functional roles of p68 in cyst formation in ADPKD have not been explored. In this study, we address a key concern regarding the mechanisms for the transcriptional regulation of the *Pkd1* gene. We found that the *Pkd1* gene is a target of p68 since 1) knockdown p68 markedly increased *Pkd1* gene expression in mIMCD3 cells; and 2) p68 bound to the *Pkd1* promoter (Figure 1). We identified a highly conserved region of p68 binding site in *Pkd1* promoters from different species. It was previously reported that p53 binds to the human *PKD1* proximal promoter within 200 bp of the transcription start site, causing transcriptional repression and represses *Pkd1* gene expression in mice and cultured cells 21. Our results presented here show that p68 interacts with p53 as examined by co-IP analysis (Figure 2). Moreover, compared the binding site of p68 and p53, we identified the upstream regulatory sequence from -209 to -65 bp region in the *Pkd1* promoter is an importance transcription factors binding site (Figure 2). These data suggest that p68 and p53 form a complex to repress the transcription of the *Pkd1* gene.

We have also determined that p68 regulates *Pkd1* gene transcription under stress stimuli. Evidence has suggested that there is reduced antioxidant enzyme protection and increased oxidative stress in ADPKD 31. Oxidative stress has been linked to molecular mechanisms associated with DNA damage and chromosomal instability, which might increase the rate of second hits or somatic mutations and thereby the severity of PKD 31. Previous studies have demonstrated that further postnatal reductions in *PKD1* (or *PKD2*) dose are required for kidney cyst formation, which can be induced by DNA damage, and activation of the DNA damage response pathway. DNA damage can be induced by high levels of H_2_O_2_ which has been shown to result in the silencing of tumor suppressor genes and uncontrolled cell growth. Previous study showed that p68 plays an important role in the p53 response to DNA damage, especially for the p53 transcriptional activity 9. We found that treatment with H_2_O_2_ decreased the expression of *Pkd1* in a p68 dependent manner, while knockdown of p68 could restore *Pkd1* expression (Figure 3). Our results suggest a scenario in ADPKD in which DNA damage results in increased oxidative stress, and oxidative stress increases the expression of p68 to further inhibit the expression of *PKD1* and promote cyst growth.

Dysregulation of miRNA expression and function has been shown to promote repression of PKD gene expression and cyst growth in ADPKD animal models 14. It has been shown that miR-17∼92 promotes cystic renal epithelial cell proliferation and cyst growth through posttranscriptional repression of the *Pkd1* and* Pkd2* genes 14. The 3′-UTR of mouse *Pkd1* mRNA contains two miR-200b/c and miR-429 binding sites, and the binding of miR-200 to these two sites represses the translation of *Pkd1* mRNA 14, 32. Bioinformatics analysis and dual luciferase assays have confirmed that *PKD1* mRNA is a direct target of miR-4787-5p. These studies suggested that miRNAs play an important role in PKD gene posttranscriptional regulation. In addition, miR-182-5p promotes cystogenesis in ADPKD via actin cytoskeleton rearrangement. However, the upstream regulators of the expression and maturation of PKD-associated miRNAs are unclear. It has been found that mice lacking Dgcr8, a key enzyme which interacts with Drosha in regulating miRNA biogenesis, results in hydronephrosis, kidney cysts, and progressive renal failure 33. An association of p68 with Drosha points to its involvement in miRNA maturation and biogenesis 34, which is also supported by observations that 1) p68 regulates the biogenesis of Let-7 miRNA 13; 2) p68 is required for the maturation of miR-125b, which in turn targets RNA-binding proteins (RBPs) to mRNA transcripts for degradation 35; and 3) p68 regulates miRNA (e.g., miR-15a) biogenesis in response to DNA damage and apoptosis 36. Based on these studies, we hypothesize that p68 is an upstream regulator of the expression and maturation of PKD-associated miRNAs. To support this notion, we found that p68 interacts with Drosha (Figure 2B, Figure 4A) in renal epithelial cells, and knockdown of p68 decreases the expression of miR-17, miR-200c and miR-182-5p in renal epithelial cells (Figure 4B). We further found that p68 is an upstream regulator of the maturation from pri-miR-17, pri-miR-200c and pri-miR-182 to pre-miR-17, pre-miR-200c and pre-miR-182 (Figure 4C and 4D), and this process requires Drosha, which formed a complex with p68 (Figure 2B, Figure 4A). In addition, we found that miR-182-5p represses *Pkd1* mRNA and PC1 protein expression in renal epithelial cells (Figure 4E to 4G). Our results suggest that p68 is a key upstream regulator of the expression of PKD-associated miRNAs and that it represses *Pkd1* expression via miR-182-5p mediated cleavage of *Pkd1* mRNA (Figure 9).

p68 plays a multifunctional role in a number of diseases, including obesity, viral infection, myotonic dystrophies and cancer 12. In addition to regulating the expression of the *Pkd1* gene in renal epithelial cells, we found that p68 was upregulated in cystic renal epithelial cells and cyst lining epithelia in *Pkd1* mutant mouse kidneys and ADPKD kidneys (Figure 5). This result suggests a role of p68 in regulating cyst development. p68 is well known as a bifunctional protein in regulating cell proliferation and fibrosis 6. It was reported that knockdown of p68 resulted in growth inhibition and cell death of prostate cancer cells and that p68-promoted glioma cell growth was mediated by activation of the ERK signaling pathway 37. p68 is also important for the DNA damage-induced G1/S cell cycle checkpoint 9. Activation of ERK, mTOR, Rb and Cyclin D1 has been associated with PKD 1. We found that knockdown of p68 decreased the phosphorylation of ERK, S6, and Rb, and the expression of Cyclin D1 in *Pkd1*-mutant PN24 cells (Figure 6G) and *Pkd1* null MEK cells (Figure S1G), suggesting that p68 can regulate the activation of PKD-associated signaling pathways to regulate cystic renal epithelial cell proliferation. In addition, upregulation of p68 increased the expression of fibronectin and α-SMA. We also found that knockdown of p68 decreased the mRNA levels of fibronectin, α-SMA and collagen-1 in *Pkd1*-mutant PN24 cells and *Pkd1* null MEK cells (Figure 7), which suggested that upregulation of p68 might promote renal fibrosis by upregulating fibrotic markers in ADPKD. Furthermore, we confirmed that knockdown of p68 delayed cyst growth in the mouse IMCD cell 3D cultures. Our study provides evidence that p68 regulates multiple physiological and pathological processes in a cellular context-dependent manner in cystic renal epithelial cells.

In sum, we have identified p68 as a novel and key regulator of ADPKD. We found that p68 negatively regulates the expression of the *Pkd1* gene by binding to its promoter in association with p53 to repress its transcription and by regulating the expression and maturation of PKD-associated miRNAs together with Drosha to post-transcriptionally regulate the cleavage and inactivation of *Pkd1* mRNA. Oxidative stress not only induced DNA damage but also increased the expression of p68, resulting in an inhibition of *Pkd1* expression. In addition, p68-mediated activation of the PKD-associated pathways, ERK, mTOR, Rb and TGF-β1, should increase cystic renal epithelial cell proliferation and renal fibrosis, resulting in cystogenesis. Given that p68 is overexpressed in cystic renal epithelial cells and tissues and knockdown of p68 delays cyst growth in 3D cultures, it may be a promising novel therapeutic target for ADPKD treatment once an inhibitor of p68 becomes available.

## Supplementary Material

Supplementary figures.

## Figures and Tables

**Table 1:** Primers used for quantitative real time PCR and ChIP-qPCR

Gene name	Forward (5'- 3')	Reverse (5'- 3')
*P68*	TTCTGATTGCTACCGATGTGG	GTGTATGCTGTGCCTGTTTTG
*P53*	ATGGCCATCTACAAGAAGTCACAG	ATCGGAGCAGCGCTCATG
*Pkd1*	CCCCGAATGTGGTTTCTATGG	GCCGTCCGATGTATGACTGC
*Pkd2*	GGGCAGCTTTCATAGACTTCTC	AGCGGATCAGTTTTACAGGC
*Actin*	AAGAGCTATGAGCTGCCTGA	TACGGATGTCAACGTCACAC
*TGF-β1*	CTGCTGACCCCCACTGATAC	AGCCCTGTATTCCGTCTCCT
*Fibronectin*	CTTTGGCAGTGGTCATTTCAG	ATTCTCCCTTTCCATTCCCG
*mPkd1 promoter*	CGGGAAAGCAGGGTGAATAG	CAGGCTTGAAGGTGGAGG
*mPkd1 promoter-NC*	TGGCTACACAAAGAAACCCTG	AACTCACTTTGTAGACCAGGC
*hPkd1 promoter*	AAATAGCTCGTGCGCCTC	CGATGAGGGACTGGCATC
*hPkd1 promoter-NC*	CAGGTTGGTCTCGAACTCTTG	ACCTGTAATCCCAGCACTTTG

## Abbreviations

ADPKD: Autosomal dominant polycystic kidney disease
PC1: polycystin-1
DDX5: DEAD-box protein 5
mIMCD3: Mouse inner-medullary collecting duct 3
RCTE: renal cortical tubular epithelia
DBA: Dolichos Biflorus Agglutinin
LTL: Lotus Tetragonolobus Lectin
ChIP: chromatin immunoprecipitation
qRT-PCR: quantitative real time polymerase chain reaction
pre-miR: precursor microRNA
pri-miR: primary microRNA
MEK: mouse embryonic kidney
PCNA: proliferating cell nuclear antigen
α-SMA: α-smooth muscle actin
Transforming growth factor-beta 1: TGF-β1
