# Supplementary material for: RNA helicase p68 inhibits the transcription and post-transcription of Pkd1 in ADPKD
Source: Theranostics. 2020 Jul 9;10(18):8281–97. doi: 10.7150/thno.47315 (PMC7381742; doi:10.7150/thno.47315)
Supplement: Supplementary file 1 — Supplementary figures. [file thnov10p8281s1.pdf]

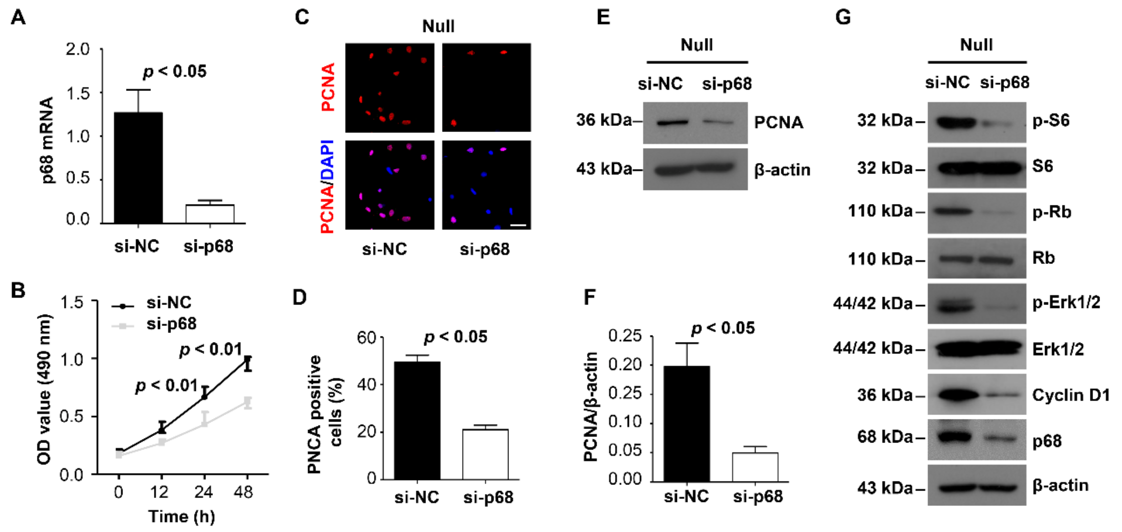

Supplemental Figure 1. p68 promotes *Pkd1* null MEK cells proliferation by activating ERK, mTOR, and Rb pathways. (**A**) The expression of p68 was examined with qRT-PCR analysis in *Pkd1* null MEK cells transfected with p68 siRNA or control siRNA for 48 h. (**B**) Cell growth of *Pkd1* null MEK cells transfected with p68 siRNA or control siRNA was determined by MTT assay. (**D** to **F**) The expression of PCNA in *Pkd1* null MEK cells transfected with p68 siRNA or control siRNA for 48 h was examined with PCNA staining (**C, D**) and Western blotting (**E, F**). Scale bars, 50 µm. (**G**) Western blot analysis of the expression of Cyclin D1 and the phosphorylation of ERK, S6 and Rb as well as the total levels of these proteins in *Pkd1* null MEK cells transfected with p68 siRNA or control siRNA for 48 h.

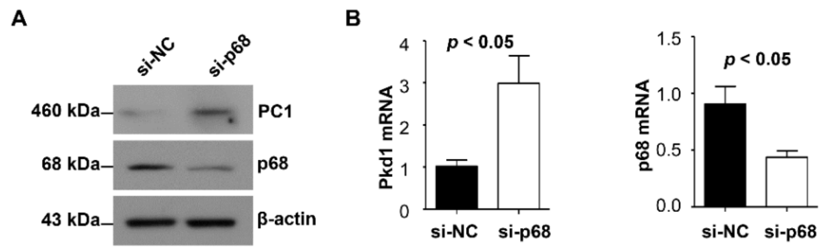

Supplemental Figure 2. Knockdown of p68 delays spheroid (cyst) growth in mIMCD3 cell 3D cultures. The expression of *Pkd1* and p68 was examined with Western blot (**A**) and qRT-PCR (**B**) analysis in 3D cultures of mIMCD3 cells transfected with p68 siRNA or negative control siRNA.
